# Factors Associated with Invasive Mechanical Ventilation Requirement in Older ICU Patients: The Roles of Frailty, Comorbidity, and Acute Illness Severity

**DOI:** 10.3390/biomedicines14092113

**Published:** 2026-09-19

**Authors:** Maşallah Çakırer, Ahmet Düzgün

**Affiliations:** Department of Intensive Care, Gazi Yaşargil Training and Research Hospital, University of Health Sciences, 21070 Diyarbakır, Türkiye; a.duzgun47@gmail.com

**Keywords:** frailty, intensive care unit, mechanical ventilation, SOFA, comorbidity, elderly

## Abstract

**Background/Objectives**: Invasive mechanical ventilation (IMV) in older intensive care unit (ICU) patients represents a critical turning point in disease trajectory. While acute illness severity is a key determinant of IMV, the independent contributions of frailty and comorbidity remain uncertain. This study evaluated whether integrating geriatric domains with acute physiological parameters provides additional information about the risk of IMV requirement, beyond acute severity alone. **Methods**: In this single-center retrospective cohort study, patients aged ≥65 years admitted to a tertiary ICU between January 2024 and December 2025 were included. Variables obtained within the first 24 h included Clinical Frailty Scale (CFS), Charlson Comorbidity Index (CCI), Sequential Organ Failure Assessment (SOFA), Acute Physiology and Chronic Health Evaluation II (APACHE II), and laboratory parameters. The primary outcome was IMV requirement. Multivariable logistic regression models were constructed, and performance was assessed using discrimination (AUC) and calibration. **Results**: Among 285 patients (median age 77 years [IQR 71–86]), 161 (56.5%) required IMV. These patients had higher frailty, comorbidity burden, and illness severity on univariable comparison. In multivariable analysis, SOFA score showed the strongest independent association with IMV requirement (adjusted OR 1.91; 95% CI 1.63–2.23; *p* < 0.001), followed by serum lactate (adjusted OR 1.30; 95% CI 1.08–1.58; *p* = 0.006). Older age was independently associated with lower odds of IMV (adjusted OR 0.96 per year; 95% CI 0.92–1.00; *p* = 0.034). Neither frailty (CFS) nor comorbidity burden (CCI) remained independently associated after adjustment. The final SOFA- and APACHE II-based models demonstrated AUCs of 0.899 (95% CI 0.864–0.933) and 0.911 (95% CI 0.879–0.944), respectively. **Conclusions**: Acute organ dysfunction and early lactate remain the dominant determinants of IMV requirement in older ICU patients. Neither comorbidity burden nor frailty retained an independent association once acute severity was accounted for; instead, advancing age was independently associated with a lower likelihood of IMV, a finding that may reflect unmeasured treatment-limitation decisions and warrants further investigation. These findings suggest that frailty and comorbidity indices should not be used in isolation as triggers for ventilatory decision-making and instead support a framework centered on acute severity and perfusion markers, with age considered mainly as a marker of care-goal context.

## 1. Introduction

Population aging has dramatically increased the proportion of older adults admitted to intensive care units (ICUs), where clinical trajectories are often complex, heterogeneous, and marked by reduced physiological reserve [1]. Compared with younger patients, older ICU patients display greater variability in comorbidities, functional status, and vulnerability to acute stressors, making early identification of those at risk for clinical deterioration a persistent challenge in geriatric critical care. This has profound implications for prognosis, resource allocation, and shared decision-making processes [2,3].

Invasive mechanical ventilation (IMV) remains one of the most invasive and consequential interventions in the ICU, particularly in older adults. Its initiation is typically a marker of acute deterioration rather than a primary therapeutic goal. In this population, IMV is associated with high rates of complications—including ventilator-associated pneumonia, delirium, critical illness myopathy, prolonged ICU and hospital stays—and substantially increased short- and long-term morbidity, mortality, and healthcare burden [4,5,6]. Notably, not all elderly ICU admissions progress to require IMV; a considerable proportion complete their ICU course without invasive ventilatory support. Elucidating the factors that distinguish patients who will require IMV from those who will not is therefore of high clinical relevance, yet this question remains incompletely addressed in contemporary geriatric cohorts [7].

Conventional severity-of-illness scoring systems, such as the Acute Physiology and Chronic Health Evaluation II (APACHE II) and Sequential Organ Failure Assessment (SOFA), effectively quantify acute physiological derangements and are widely employed for risk stratification. However, these tools capture only limited aspects of the biological vulnerability inherent to aging. In contrast, frailty—reflecting cumulative deficits in physiological reserve and stress tolerance—and overall comorbidity burden represent core geriatric dimensions that may modulate critical illness trajectories beyond acute severity alone [8].

Emerging evidence indicates that frailty is prevalent among critically ill older adults (often 30–50% in cohorts aged ≥65 years) and is consistently linked to adverse outcomes, including higher mortality, prolonged length of stay, and increased resource utilization [9,10]. Several studies have reported associations between frailty and greater duration of mechanical ventilation or weaning difficulties [5,11], yet its independent role in predicting the requirement for IMV—after accounting for acute organ dysfunction—remains controversial. While some investigations indicate frailty amplifies the likelihood of respiratory failure and ventilatory support [12], others find that this association diminishes or disappears after multivariable adjustment for acute illness severity, with no significant difference in IMV utilization between frail and non-frail patients in meta-analyses [9]. This inconsistency highlights a key knowledge gap: the extent to which baseline geriatric vulnerability drives the need for IMV, versus serving primarily as a predisposing factor that potentiates acute insults [13].

Therefore, the present study aimed to compare the clinical characteristics of older ICU patients (≥65 years) who required invasive mechanical ventilation at any point during their ICU stay with those who did not, and to identify factors independently associated with IMV requirement. By integrating acute illness severity measures (APACHE II and SOFA scores) with geriatric-specific domains—including frailty assessed by the Clinical Frailty Scale (CFS) and cumulative comorbidity burden quantified by the Charlson Comorbidity Index (CCI)—we sought to provide a more comprehensive and nuanced characterization of patients at heightened risk for invasive ventilatory support during critical illness.

## 2. Materials and Methods

### 2.1. Study Design and Setting

This single-center, retrospective observational cohort study was conducted in the tertiary-level multidisciplinary intensive care unit. The study adhered to the Strengthening the Reporting of Observational Studies in Epidemiology (STROBE) guidelines for cohort studies.

### 2.2. Study Population

All consecutive patients aged ≥65 years admitted to the ICU between January 2024 and December 2025 were screened for eligibility. The inclusion criteria were (1) age ≥65 years at ICU admission and (2) ICU length of stay ≥ 24 h. For patients with multiple ICU admissions during the study period, only the first admission was considered.

Patients were excluded if they met any of the following criteria: (1) ICU length of stay < 24 h; (2) repeat ICU admission during the study period; (3) receipt of long-term invasive mechanical ventilation (>30 days) prior to ICU admission; (4) elective postoperative admission without acute critical illness; or (5) missing essential baseline data required for the primary analysis.

### 2.3. Definition of Study Groups

Patients were dichotomized based on the requirement for invasive mechanical ventilation (IMV) at any time during the ICU stay:

• IMV Group: Patients who received endotracheal intubation and invasive ventilatory support (including those intubated on or after ICU admission).
• Non-IMV Group: Patients who did not receive IMV at any point during ICU hospitalization.

### 2.4. Data Collection

All predictor variables were retrospectively extracted from the institutional electronic medical record (EMR) system, with all relevant data limited to the first 24 h following ICU admission. This time-restricted approach was employed to standardize predictor ascertainment and ensure that variables reflected the patient’s early clinical status. However, because a subset of patients were already intubated on or shortly after ICU admission, this approach does not fully eliminate temporal ambiguity between predictor measurement and outcome occurrence.

Collected variables encompassed demographics (age and sex), admission diagnosis (categorized as medical, surgical, sepsis/septic shock, respiratory failure, or other relevant groups), and comorbidity burden, which was quantified using the Charlson Comorbidity Index (CCI) derived from ICD-10 codes and supplementary chart review. Frailty was assessed using the Clinical Frailty Scale (CFS; score range 1–9), scored by the admitting intensivist or multidisciplinary ICU team. The CFS evaluation was based on the patient’s pre-morbid functional status (typically referring to the period 2 weeks prior to the acute illness) and incorporated information from the patient, relatives, caregivers, and existing medical records, in accordance with standard methodology [8]. Notably, CFS scores were documented prospectively as part of routine ICU admission assessments rather than retrospectively assigned for this study. Acute illness severity was evaluated using the Acute Physiology and Chronic Health Evaluation II (APACHE II) and Sequential Organ Failure Assessment (SOFA) scores, calculated according to their established definitions. Briefly, the CCI is a weighted index summing 19 age- and comorbidity-related conditions, with higher scores indicating greater cumulative comorbidity burden and higher predicted long-term mortality risk. The CFS is a 9-point clinician-rated scale (1, very fit, to 9, terminally ill) capturing baseline functional reserve and vulnerability to acute stressors prior to the index illness. The SOFA score (range 0–24) quantifies the degree of dysfunction across six organ systems (respiratory, coagulation, hepatic, cardiovascular, neurological, and renal), with higher scores reflecting more severe acute organ dysfunction. The APACHE II score (range 0–71) integrates acute physiological derangement across multiple parameters with age and chronic health status to estimate risk of ICU mortality. Laboratory parameters obtained within the first 24 h included serum lactate, C-reactive protein (CRP), albumin, absolute neutrophil count, and absolute lymphocyte count, from which the neutrophil-to-lymphocyte ratio (NLR) was computed as the ratio of neutrophils to lymphocytes. Antibiotic therapy within the first 24 h was recorded as a treatment variable; it was not considered equivalent to confirmed infection, as it may reflect empiric or prophylactic use. Site-specific infection source was not systematically coded in the institutional dataset and could therefore not be reported.

Of the 326 patients initially screened, 41 were excluded: 8 for an ICU length of stay < 24 h, 12 for repeat ICU admission during the study period (identified during data verification by cross-referencing patient identifiers, with age and sex used to confirm true repeat admissions; only the earliest admission was retained), 3 for long-term invasive mechanical ventilation (>30 days) prior to ICU admission, 11 for elective postoperative admission without acute critical illness, and 7 for missing or non-computable essential baseline data (4 identified during initial screening; 3 identified during a subsequent data audit—1 missing serum lactate and 2 with a lymphocyte count of zero, precluding calculation of the neutrophil-to-lymphocyte ratio). The final analytical cohort comprised 285 patients with complete data for all variables included in the primary analysis; no imputation was performed.

### 2.5. Outcome Measure

The primary outcome was the requirement for invasive mechanical ventilation during the ICU stay, defined as initiation of positive pressure ventilation via endotracheal tube or tracheostomy at any time after ICU admission (or on admission if already intubated for acute reason).

### 2.6. Statistical Analysis

Continuous variables were assessed for normality using the Shapiro–Wilk test, along with visual inspection of histograms and Q-Q plots. Normally distributed variables were summarized as mean ± standard deviation (SD), while non-normally distributed variables were presented as median (interquartile range [IQR]). Categorical variables were expressed as counts (percentages). Group comparisons were performed using the independent samples *t*-test or Mann–Whitney U test for continuous variables, and the chi-square test or Fisher’s exact test for categorical variables, as appropriate.

Univariable logistic regression analyses were conducted to identify candidate predictors of invasive mechanical ventilation (IMV) requirement, using a liberal entry criterion (*p* < 0.10) or clinical relevance. Multivariable logistic regression models were then constructed to determine independent factors associated with IMV. To mitigate potential collinearity between acute severity scores, separate models were developed incorporating either the APACHE II or SOFA score (alongside age, sex, Charlson Comorbidity Index [CCI], Clinical Frailty Scale [CFS], serum lactate, albumin, C-reactive protein [CRP], and neutrophil-to-lymphocyte ratio [NLR]). Variable selection employed backward elimination based on the likelihood ratio test, with predictors retained at *p* < 0.05.

Model performance was assessed through the following metrics: discrimination via the area under the receiver operating characteristic curve (AUC) with 95% confidence intervals (Hanley–McNeil method, as implemented in the SPSS ROC procedure); calibration via the Hosmer–Lemeshow goodness-of-fit test, calibration plots (observed vs. predicted probability deciles), and Brier score; and explanatory power via Nagelkerke pseudo-R^2^.

All tests were two-sided, with statistical significance defined as *p* < 0.05. Analyses were performed using IBM SPSS Statistics version 24 (IBM Corp., Armonk, NY, USA).

### 2.7. Ethical Considerations

The study protocol was reviewed and approved by the Clinical Research Ethics Committee of Diyarbakır Gazi Yaşargil Training and Research Hospital, University of Health Sciences, Diyarbakır, Türkiye (approval No.: 64; date: 12 February 2026). The requirement for informed consent was waived owing to the retrospective nature of the study and the use of anonymized data. The study was conducted in accordance with the Declaration of Helsinki and applicable national regulations.

## 3. Results

### 3.1. Patient Screening and Study Population

**Figure 1:**
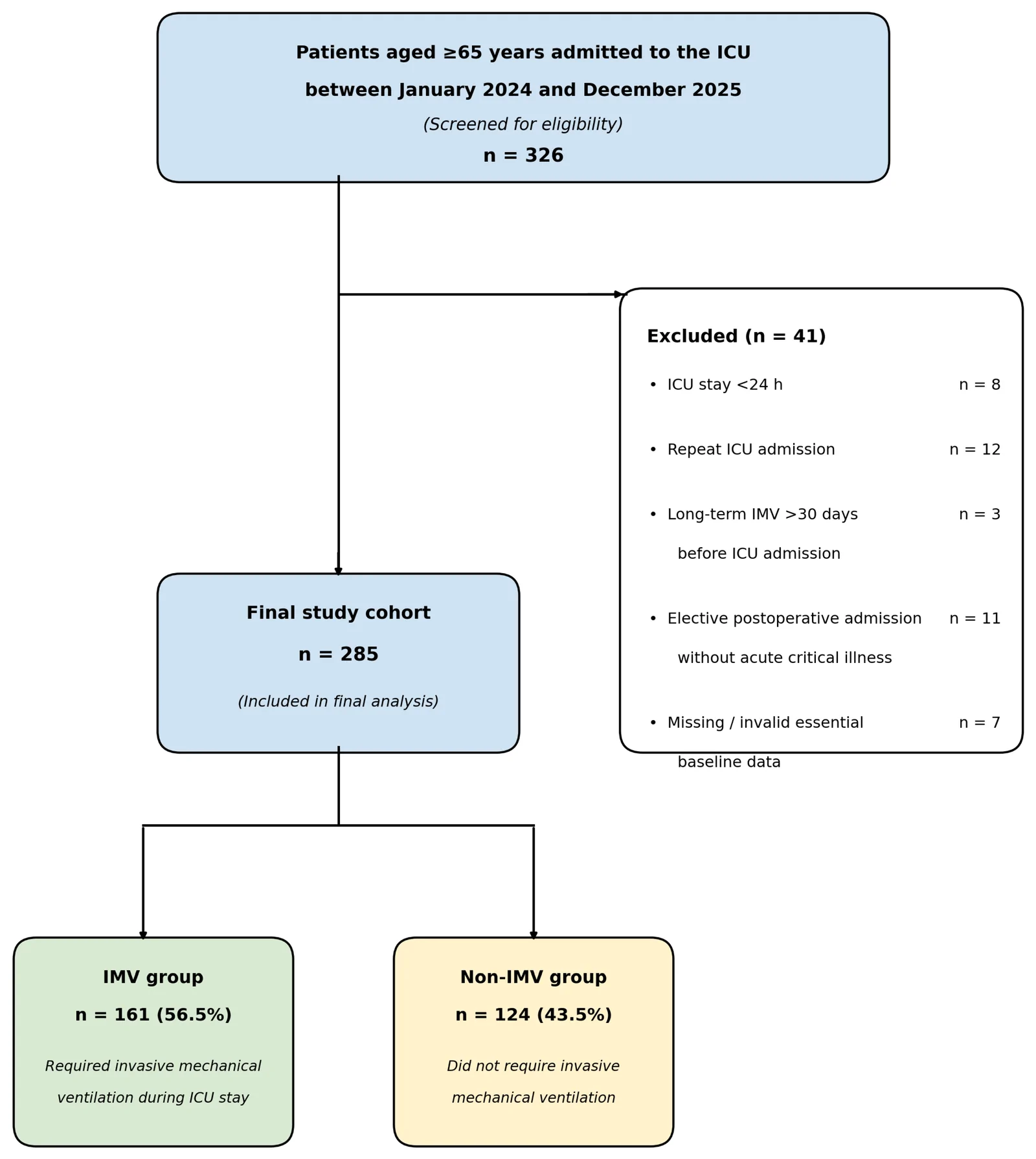
Flow diagram of patient screening, exclusions, and final study group allocation. ICU, intensive care unit; IMV, invasive mechanical ventilation.

A total of 326 patients aged ≥65 years admitted to the ICU between January 2024 and December 2025 were screened for eligibility. Of these, 41 patients were excluded: 8 because of an ICU length of stay < 24 h, 12 because of repeat ICU admission during the study period, 3 because of long-term invasive mechanical ventilation (>30 days) prior to ICU admission, 11 because of elective postoperative admission without acute critical illness, and 7 because of missing or non-computable essential baseline data. The final study cohort therefore comprised 285 patients. Of these, 161 (56.5%) required invasive mechanical ventilation (IMV) during their ICU stay, whereas 124 (43.5%) did not require IMV (Figure 1).

### 3.2. Comparison of Clinical Characteristics Between IMV and Non-IMV Groups

Baseline demographic characteristics were comparable between groups, with no significant difference in median age (76 years [IQR 70–84] in IMV vs. 79 years [IQR 72–86] in non-IMV; *p* = 0.198) or sex distribution (male 50.3% vs. 45.2%; *p* = 0.388).

Markers of biological vulnerability and acute illness severity differed markedly. Patients requiring IMV had significantly higher frailty (Clinical Frailty Scale median 7 [IQR 6–8] vs. 6 [IQR 5–7]; *p* < 0.001) and greater comorbidity burden (Charlson Comorbidity Index median 7 [IQR 6–7] vs. 6 [IQR 5–6]; *p* < 0.001). Acute physiological derangement was substantially greater in the IMV group, as evidenced by higher SOFA scores (median 10 [IQR 8–12] vs. 5 [IQR 4–7]; *p* < 0.001) and APACHE II scores (median 24 [IQR 19–29] vs. 12 [IQR 10–16]; *p* < 0.001). Early laboratory parameters also showed pronounced differences: serum lactate was elevated (median 2.8 mmol/L [IQR 1.9–6.0] vs. 1.8 mmol/L [IQR 1.4–2.6]; *p* < 0.001), C-reactive protein was higher (median 70 mg/L [IQR 15–145] vs. 27 mg/L [IQR 7–72]; *p* < 0.001), and serum albumin was lower (median 27 g/L [IQR 23–34] vs. 32 g/L [IQR 28–36]; *p* < 0.001) in the IMV group. The neutrophil-to-lymphocyte ratio did not differ significantly between groups (median 7.6 [IQR 4.3–13.8] vs. 9.2 [IQR 5.0–14.8]; *p* = 0.333).

These comparisons are detailed in Table 1.

### 3.3. Case-Mix, Comorbidity Profile, and Outcome Indicators by IMV Group

The admission diagnosis category differed markedly between groups (*p* < 0.001): the IMV group was predominantly composed of medical (46.0%) and sepsis/septic shock (31.7%) admissions, whereas the non-IMV group was predominantly composed of non-elective postoperative surgical admissions (46.0%). On univariable comparison of comorbidity subtypes, prior stroke (19.3% vs. 9.7%; *p* = 0.038), chronic kidney disease (34.2% vs. 14.5%; *p* < 0.001), and the presence of any comorbidity (98.8% vs. 91.9%; *p* = 0.011) were more frequent in the IMV group; other individual comorbidity types, including diabetes, hypertension, coronary artery disease, congestive heart failure, atrial fibrillation, COPD, Alzheimer’s disease, peripheral arterial disease, and malignancy, did not differ significantly between groups. Chronic kidney disease and prior stroke were more frequent in the IMV group on univariable comparison. However, individual comorbidity subtypes were not entered separately into the multivariable models which incorporated only the aggregate Charlson Comorbidity Index; therefore, the independent association of these specific comorbidities with IMV requirement cannot be determined from the present analysis.

Patients requiring IMV also had markedly lower admission Glasgow Coma Scale scores, longer ICU length of stay, higher rates of renal replacement therapy and tracheostomy, more frequent antibiotic use, and substantially higher 28-day mortality (58.4% vs. 0%) than the non-IMV group. These findings are summarized in Table 2.

### 3.4. Univariable Analysis

In univariable logistic regression, variables associated with IMV requirement included higher SOFA score (*p* < 0.001), APACHE II score (*p* < 0.001), Clinical Frailty Scale (*p* < 0.001), Charlson Comorbidity Index (*p* < 0.001), serum lactate (*p* < 0.001), C-reactive protein (*p* < 0.001), and lower serum albumin (*p* < 0.001). Age (*p* = 0.261), sex (*p* = 0.389), and neutrophil-to-lymphocyte ratio (*p* = 0.365) did not meet criteria for inclusion (*p* < 0.10) on their own but were retained as candidates alongside the clinically pre-specified covariate set. These candidates were carried forward to multivariable modeling.

### 3.5. Multivariable Analysis of Factors Associated with IMV Requirement

Separate multivariable logistic regression models were constructed incorporating either SOFA or APACHE II, along with age, sex, Charlson Comorbidity Index, Clinical Frailty Scale, serum lactate, albumin, C-reactive protein, and neutrophil-to-lymphocyte ratio. Backward elimination (likelihood ratio test, *p* < 0.05 retention) was applied.

In the SOFA-based model, higher SOFA score showed the strongest independent association with IMV requirement (adjusted OR per 1-point increase: 1.91, 95% CI 1.63–2.23; *p* < 0.001), followed by serum lactate (adjusted OR per 1 mmol/L increase: 1.30, 95% CI 1.08–1.58; *p* = 0.006). Age was retained as an independently associated factor but in the opposite direction to expectation: each additional year of age was associated with lower odds of IMV (adjusted OR 0.96, 95% CI 0.92–1.00; *p* = 0.034). Sex, Charlson Comorbidity Index, Clinical Frailty Scale, albumin, C-reactive protein, and neutrophil-to-lymphocyte ratio did not reach the retention threshold and were removed during backward elimination.

The final SOFA-based model demonstrated an AUC of 0.899 (95% CI 0.864–0.933), a Nagelkerke pseudo-R^2^ of 0.591, and a Brier score of 0.130. The Hosmer–Lemeshow test showed some evidence of imperfect calibration (χ^2^ = 18.6, df = 8, *p* = 0.017), which is discussed as a limitation below.

Multivariable findings for the SOFA-based model are summarized in Table 3.

In the APACHE II-based model, APACHE II remained the factor most strongly associated with IMV requirement (adjusted OR 1.38, 95% CI 1.28–1.50; *p* < 0.001), together with age (adjusted OR 0.94, 95% CI 0.90–0.98; *p* = 0.008), lactate (adjusted OR 1.20, 95% CI 1.01–1.44; *p* = 0.037), and, unlike the SOFA-based model, neutrophil-to-lymphocyte ratio (adjusted OR 1.04, 95% CI 1.00–1.07; *p* = 0.043). This model showed slightly better discrimination (AUC 0.911, 95% CI 0.879–0.944) and good calibration (Hosmer–Lemeshow χ^2^ = 1.28, df = 8, *p* = 0.996), with a Nagelkerke pseudo-R^2^ of 0.629 and Brier score of 0.119. Full results are presented in Table 4.

**Table 3:** Univariable and Multivariable Logistic Regression Analysis of Factors Associated with Invasive Mechanical Ventilation Requirement—SOFA-Based Model.

Variable	Univariable OR (95% CI)	Univariable *p*	Adjusted OR (95% CI)	Adjusted *p*
SOFA score (per 1-point increase)	1.96 (1.69–2.28)	<0.001	1.91 (1.63–2.23)	<0.001
Serum lactate (per 1 mmol/L increase)	1.56 (1.31–1.87)	<0.001	1.30 (1.08–1.58)	0.006
Age (per 1-year increase)	0.98 (0.96–1.01)	0.261	0.96 (0.92–1.00)	0.034
Charlson Comorbidity Index (per 1-point increase)	1.44 (1.21–1.70)	<0.001	— (removed)	—
Clinical Frailty Scale (per 1-point increase)	1.37 (1.18–1.60)	<0.001	— (removed)	—
Serum albumin (per 1 g/L increase)	0.93 (0.90–0.96)	<0.001	— (removed)	—
C-reactive protein (per 1 mg/L increase)	1.01 (1.00–1.01)	<0.001	— (removed)	—
Sex (male vs. female)	1.23 (0.77–1.97)	0.389	— (removed)	—
Neutrophil-to-lymphocyte ratio (per 1-unit increase)	1.01 (0.99–1.03)	0.365	— (removed)	—

Final SOFA-based model: AUC 0.899 (95% CI 0.864–0.933); Nagelkerke pseudo-R^2^ 0.591; Brier score 0.130; Hosmer–Lemeshow χ^2^ = 18.6, df = 8, *p* = 0.017. Variable selection by backward elimination (likelihood ratio test, retention threshold *p* < 0.05). Abbreviations: CI, confidence interval; OR, odds ratio; SOFA, Sequential Organ Failure Assessment.

**Figure 2:**
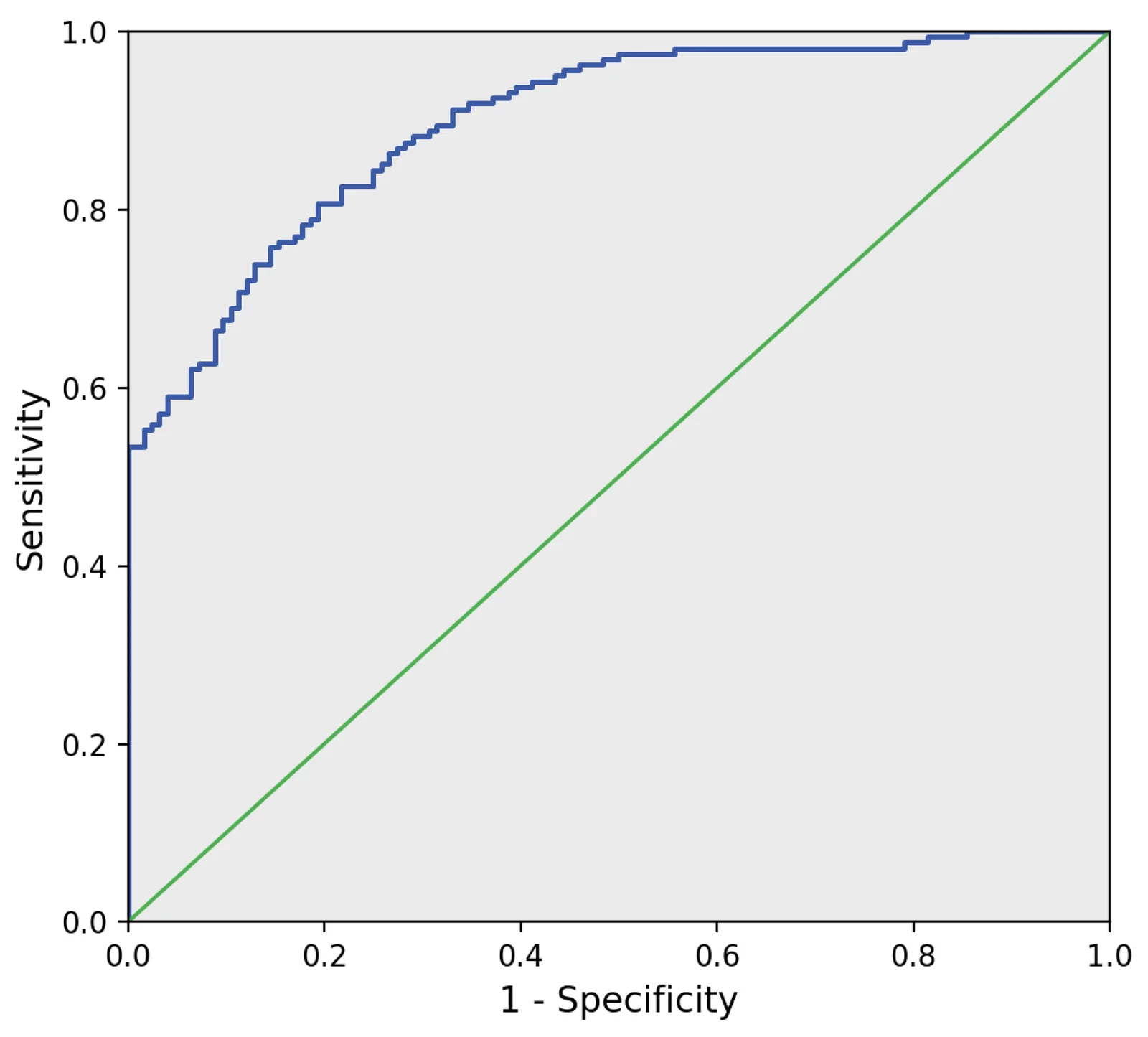
Receiver operating characteristic (ROC) curve of the final SOFA-based multivariable model predicting invasive mechanical ventilation. The blue line represents the model’s ROC curve, and the green diagonal line represents the line of no discrimination (reference line, AUC = 0.5). The model demonstrated an AUC of 0.899 (95% CI 0.864–0.933).

As shown in Figure 2, the final SOFA-based multivariable model demonstrated an AUC of 0.899 (95% CI 0.864–0.933). SOFA alone yielded an AUC of 0.883 (95% CI 0.845–0.921), slightly lower than that of the multivariable model.

**Table 4:** Multivariable Logistic Regression Analysis of Factors Associated with Invasive Mechanical Ventilation Requirement—APACHE II-Based Model.

Variable	Adjusted OR (95% CI)	Adjusted *p*
APACHE II score (per 1-point increase)	1.38 (1.28–1.50)	<0.001
Age (per 1-year increase)	0.94 (0.90–0.98)	0.008
Serum lactate (per 1 mmol/L increase)	1.20 (1.01–1.44)	0.037
Neutrophil-to-lymphocyte ratio (per 1-unit increase)	1.04 (1.00–1.07)	0.043

Sex, Charlson Comorbidity Index, Clinical Frailty Scale, serum albumin, and C-reactive protein were removed during backward elimination (likelihood ratio test, retention threshold *p* < 0.05). Final APACHE II-based model: AUC 0.911 (95% CI 0.879–0.944); Nagelkerke pseudo-R^2^ 0.629; Brier score 0.119; Hosmer–Lemeshow χ^2^ = 1.28, df = 8, *p* = 0.996. Abbreviations: APACHE II, Acute Physiology and Chronic Health Evaluation II; CI, confidence interval; OR, odds ratio.

## 4. Discussion

In this retrospective cohort of 285 critically ill older adults (≥65 years) admitted to a tertiary intensive care unit, more than half (56.5%) required invasive mechanical ventilation (IMV) at some point during their ICU stay. After comprehensive adjustment for acute physiological derangement, SOFA score and early serum lactate remained the factors most strongly and independently associated with IMV requirement, regardless of whether SOFA or APACHE II was used as the acute-severity anchor. Contrary to our initial hypothesis, neither the Charlson Comorbidity Index (CCI) nor the Clinical Frailty Scale (CFS) retained independent significance once acute severity and lactate were accounted for, with both removed during backward elimination before the final model was reached. Instead, age itself emerged as an independently associated factor, but in an inverse direction to what is conventionally assumed: each additional year of age was associated with modestly lower odds of receiving IMV (adjusted OR 0.96 per year in the SOFA-based model; 0.94 in the APACHE II-based model). This pattern was consistent across both severity-adjustment strategies, which argues against a simple collinearity artifact specific to one model.

Our results help reconcile, and in part sharpen, the existing controversy surrounding frailty and ventilatory support in older ICU patients. On univariable comparison, frail patients (higher CFS) and those with greater comorbidity burden (higher CCI) were markedly over-represented in the IMV group, mirroring numerous prior reports. However, once SOFA (or APACHE II) and lactate were entered into the model, both associations were fully attenuated. This is consistent with meta-analyses reporting conflicting or null associations between frailty and the initiation of IMV after multivariable adjustment for acute illness severity [9,11,14], and extends this observation to comorbidity burden as well [15]: a substantial unadjusted CCI effect (univariable OR 1.44) was no longer independently associated with IMV after adjustment for acute severity and perfusion status. Taken together, these findings suggest that for the specific binary outcome of IMV requirement—as distinct from weaning success, ventilator-free days, or long-term functional recovery, outcomes on which frailty is more consistently shown to exert an independent effect [5,10]—acute organ dysfunction and tissue perfusion appear to be more strongly associated with IMV requirement, while baseline vulnerability indices contribute comparatively little incremental discriminatory information once these are known [16].

The independent, inverse association between age and IMV requirement is, in our view, the most clinically important finding of this study and warrants careful interpretation. We do not believe it reflects a genuine reduction in physiological need for ventilatory support in the oldest patients; if anything, biological reserve declines with age. A more plausible explanation is selective limitation of invasive interventions in very old patients—a phenomenon well documented in European ICU cohorts, where physician attitudes toward escalation of care, and the likelihood of a treatment ceiling being set, are known to shift with advancing age and perceived frailty [2,3]. Elevated admission lactate, which remained independently associated with IMV requirement after adjustment, together with the lower albumin levels observed in the IMV group on univariable comparison further underscore the prominent role of tissue hypoperfusion and a systemic inflammatory-catabolic state among the factors associated with IMV requirement in this population [17,18]; the lactate association was independent of the patient’s age or chronic disease burden, whereas the albumin difference did not persist after adjustment for acute severity. Because our dataset did not capture do-not-intubate orders, documented goals-of-care discussions, or family preferences at admission, we cannot directly test the treatment-limitation hypothesis, and it should be regarded as hypothesis-generating rather than confirmed. Collectively, these findings suggest a hierarchical model of risk in geriatric critical illness in which acute physiological derangement and perfusion failure set the proximate “tipping point” for IMV, while age and baseline vulnerability act—if at all—as modifiers of the threshold at which clinicians and families choose to intervene, rather than as independent drivers of physiological need [19].

**Figure 3:**
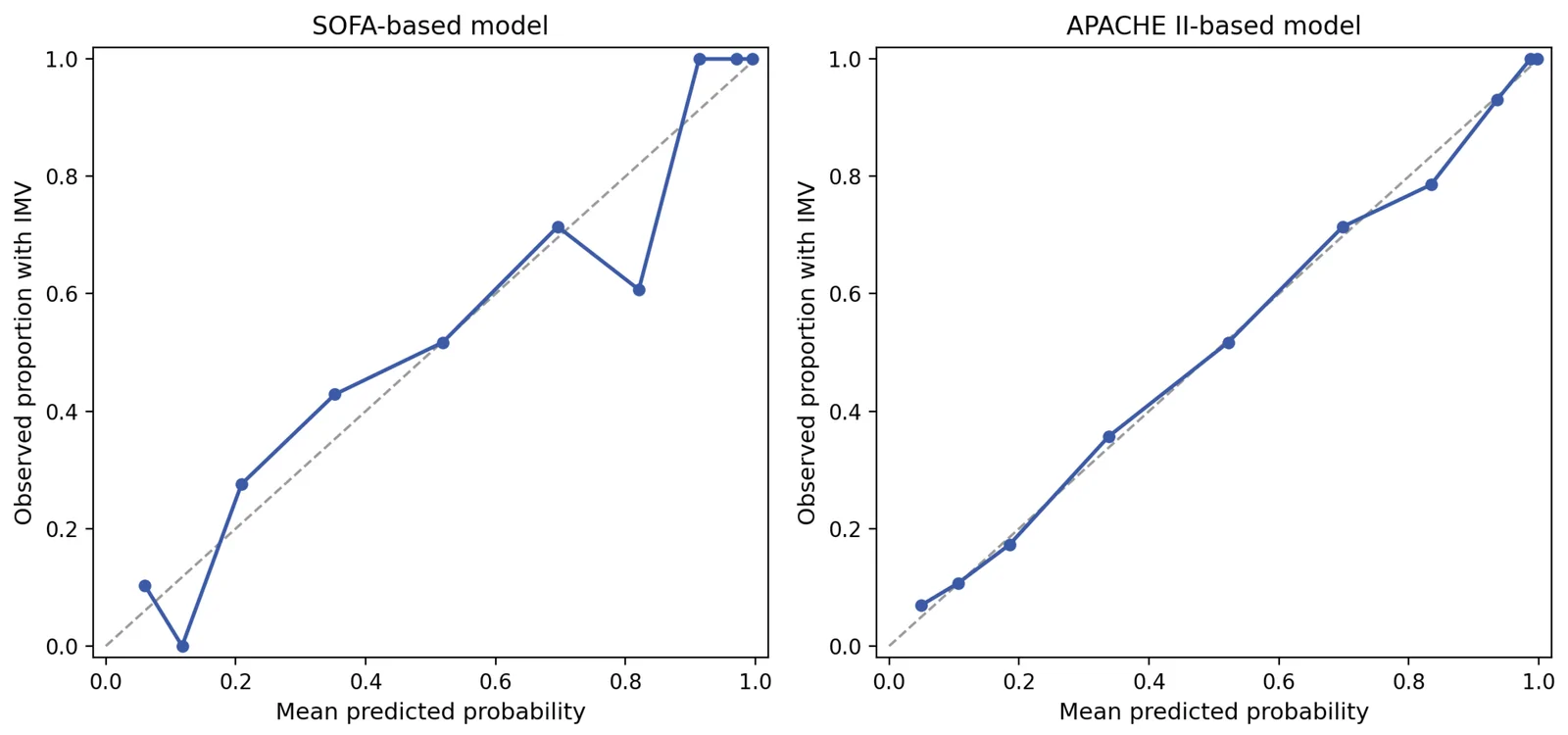
Calibration plots (observed vs. predicted probability of IMV, by decile of predicted risk) for the SOFA-based and APACHE II-based multivariable models. The dashed line indicates perfect calibration.

The SOFA-based model showed excellent discrimination (AUC 0.899, 95% CI 0.864–0.933) but some evidence of imperfect calibration on Hosmer–Lemeshow testing (χ^2^ = 18.6, df = 8, *p* = 0.017); the APACHE II-based model discriminated marginally better (AUC 0.911, 95% CI 0.879–0.944) and calibrated well (*p* = 0.996). This dissociation between strong discrimination and imperfect calibration in the SOFA-based model is a recognized limitation of the Hosmer–Lemeshow test when a single covariate dominates the model in a moderate-sized sample, and we report it transparently rather than presenting only the more favorable metric; calibration plots for both models (Figure 3) should be interpreted alongside this result, and the APACHE II-based model may be the more robust choice where calibrated probability estimates are needed.

Strengths of the present study include the consecutive enrollment of a real-world tertiary ICU cohort over a full two-year period, the use of prospectively recorded CFS as part of routine admission assessment (minimizing recall bias), a systematic secondary data audit that identified and excluded duplicate patient records and non-computable laboratory values prior to analysis, and the construction of separate SOFA- and APACHE II-based multivariable models to address collinearity between acute severity scores [9,14].

Several limitations must be acknowledged. First, the single-center retrospective design limits generalizability, although the tertiary multidisciplinary setting reflects the case-mix encountered in most large academic ICUs. Second, although predictor ascertainment was restricted to the first 24 h to standardize early clinical assessment and reduce temporal ambiguity between predictor measurement and outcome occurrence, residual confounding by unmeasured factors—most importantly, treatment-limitation decisions, goals-of-care discussions, and pre-ICU functional trajectory, none of which were captured in our dataset—cannot be excluded, and we consider this the most likely explanation for the inverse age association observed. In addition, because IMV could be initiated on or immediately after ICU admission, predictor values ascertained within the first 24 h—most notably the SOFA score, whose respiratory sub-component is itself partly determined by ventilatory status—may in some patients have been measured concurrently with or after IMV initiation rather than strictly beforehand; the reported associations should therefore be interpreted as factors associated with IMV requirement rather than as strictly prospective predictors of subsequent intubation. Third, the modest calibration concern identified in the SOFA-based model warrants caution when applying its point estimates to individual-level risk prediction without external validation. Fourth, external validation in a multicenter cohort is required before clinical implementation. Fifth, our institutional dataset did not systematically capture vasopressor use, use of non-invasive ventilation or high-flow nasal cannula therapy (and, consequently, the interval between such support and subsequent intubation), site-specific source of infection, or standardized measures of pre-admission functional status and dementia beyond the Clinical Frailty Scale and a binary Alzheimer’s disease diagnosis; these omissions limit our ability to characterize the trajectory toward IMV in greater granularity and should be addressed in future prospective work. Finally, we focused exclusively on the binary outcome of IMV requirement rather than downstream consequences such as weaning success, ventilator-free days, or long-term functional recovery, outcomes on which frailty is known to exert a stronger and more consistent influence.

From a clinical perspective, our findings suggest that frailty or comorbidity indices should not be used as standalone gatekeepers for IMV initiation in older ICU patients: once acute severity and lactate are known, CFS and CCI added no independent additional information beyond acute severity and lactate in this cohort. This does not diminish the value of routine CFS screening at ICU admission, which remains informative for anticipating prolonged support, complications, and long-term outcomes even where it does not predict the immediate need for intubation [5,20]. Rather, it suggests that decisions around IMV itself should be anchored primarily in acute organ dysfunction and perfusion markers, with age and frailty considered explicitly as context for goals-of-care conversations rather than as quantitative predictors of physiological need. Prospective studies that directly capture treatment-limitation decisions, alongside dynamic frailty trajectories, are warranted to test this interpretation and to determine whether structured goals-of-care discussions—rather than frailty or comorbidity scores per se—explain the lower observed rate of IMV among the oldest patients in this cohort [14,21,22].

## 5. Conclusions

In conclusion, among older adults admitted to the ICU, acute organ dysfunction and early lactate remain the dominant, independent determinants of invasive mechanical ventilation requirement. Neither comorbidity burden nor frailty provided independent additive value once acute severity was accounted for; instead, advancing age was independently—and inversely—associated with IMV, a finding that may partly reflect unmeasured treatment-limitation decisions rather than reduced physiological need. These findings caution against using frailty or comorbidity scores as standalone gatekeepers for ventilatory decisions and instead support a framework centered on acute severity and perfusion markers, interpreted alongside explicit goals-of-care discussions in the oldest patients.

## Figures and Tables

**Table 1:** Baseline Clinical and Demographic Characteristics of Older ICU Patients by Invasive Mechanical Ventilation Requirement.

Variable	IMV Group (n = 161)	Non-IMV Group (n = 124)	*p*-Value
Age, years (median, IQR)	76 (70–84)	79 (72–86)	0.198
Male sex, n (%)	81 (50.3)	56 (45.2)	0.388
Clinical Frailty Scale (median, IQR)	7 (6–8)	6 (5–7)	<0.001
Charlson Comorbidity Index (median, IQR)	7 (6–7)	6 (5–6)	<0.001
SOFA score (median, IQR)	10 (8–12)	5 (4–7)	<0.001
APACHE II score (median, IQR)	24 (19–29)	12 (10–16)	<0.001
Serum lactate, mmol/L (median, IQR)	2.8 (1.9–6.0)	1.8 (1.4–2.6)	<0.001
CRP, mg/L (median, IQR)	70 (15–145)	27 (7–72)	<0.001
Albumin, g/L (median, IQR)	27 (23–34)	32 (28–36)	<0.001
NLR (median, IQR)	7.6 (4.3–13.8)	9.2 (5.0–14.8)	0.333

Abbreviations: APACHE II, Acute Physiology and Chronic Health Evaluation II; CRP, C-reactive protein; ICU, intensive care unit; IMV, invasive mechanical ventilation; IQR, interquartile range; NLR, neutrophil-to-lymphocyte ratio; SOFA, Sequential Organ Failure Assessment. *p*-values from the Mann–Whitney U test (continuous variables) or chi-square test (sex).

**Table 2:** Case-Mix, Comorbidity Profile, and Outcome Indicators by Invasive Mechanical Ventilation Requirement.

Variable	IMV Group (n = 161)	Non-IMV Group (n = 124)	*p*-Value
***Admission Diagnosis Category, n (%)***			
Medical	74 (46.0)	19 (15.3)	<0.001 *
Sepsis/Septic shock	51 (31.7)	12 (9.7)	—
Respiratory failure	13 (8.1)	13 (10.5)	—
Surgical (non-elective postoperative)	10 (6.2)	57 (46.0)	—
Trauma	13 (8.1)	23 (18.5)	—
***Comorbidity Type, n (%)***			
Diabetes mellitus	60 (37.3)	41 (33.1)	0.542
Hypertension	97 (60.2)	87 (70.2)	0.108
Coronary artery disease	61 (37.9)	43 (34.7)	0.664
Congestive heart failure	52 (32.3)	35 (28.2)	0.542
Atrial fibrillation	32 (19.9)	20 (16.1)	0.511
COPD	34 (21.1)	30 (24.2)	0.636
Prior stroke	31 (19.3)	12 (9.7)	0.038
Alzheimer’s disease	32 (19.9)	18 (14.5)	0.307
Peripheral arterial disease	3 (1.9)	2 (1.6)	1.000
Chronic kidney disease	55 (34.2)	18 (14.5)	<0.001
Hematologic malignancy	5 (3.1)	4 (3.2)	1.000
Solid organ malignancy	19 (11.8)	7 (5.6)	0.114
≥1 comorbidity present	159 (98.8)	114 (91.9)	0.011
***Other Clinical Indicators***			
Admission GCS (median, IQR)	9 (5–13)	14 (14–15)	<0.001
IMV duration, days (median, IQR)	7 (2–22)	N/A	—
ICU length of stay, days (median, IQR)	12 (4–31)	4 (2–7)	<0.001
Renal replacement therapy, n (%)	40 (24.8)	5 (4.0)	<0.001
Tracheostomy, n (%)	32 (19.9)	0 (0.0)	<0.001
Antibiotic use, n (%)	149 (92.5)	78 (62.9)	<0.001
28-day mortality, n (%)	94 (58.4)	0 (0.0)	<0.001

Abbreviations: COPD, chronic obstructive pulmonary disease; GCS, Glasgow Coma Scale; ICU, intensive care unit; IMV, invasive mechanical ventilation; IQR, interquartile range; N/A, not applicable. *p*-values from the chi-square test or Fisher’s exact test (categorical variables, as appropriate) or the Mann–Whitney U test (continuous variables). * Admission diagnosis category was compared using a single global chi-square test across all five categories (*p* < 0.001); this global *p*-value is reported once and does not represent a separate per-category test. IMV duration is reported for the IMV group only, as this variable is not applicable to patients who did not receive IMV. Vasopressor use, non-invasive ventilation/high-flow nasal cannula use prior to intubation, site-specific infection source, and standardized functional status/dementia assessments beyond the Clinical Frailty Scale and Alzheimer’s disease diagnosis were not systematically captured in the institutional dataset and could not be reported.

## Data Availability

The data presented in this study are available from the corresponding author upon reasonable request.

## Abbreviations

The following abbreviations are used in this manuscript:

**Abbreviation**	**Definition**
APACHE II	Acute Physiology and Chronic Health Evaluation II
AUC	Area Under the Receiver Operating Characteristic Curve
CCI	Charlson Comorbidity Index
CFS	Clinical Frailty Scale
CI	Confidence Interval
CRP	C-Reactive Protein
EMR	Electronic Medical Record
ICU	Intensive Care Unit
IMV	Invasive Mechanical Ventilation
IQR	Interquartile Range
NLR	Neutrophil-to-Lymphocyte Ratio
OR	Odds Ratio
ROC	Receiver Operating Characteristic
SD	Standard Deviation
SOFA	Sequential Organ Failure Assessment
STROBE	Strengthening the Reporting of Observational Studies in Epidemiology
